# Supplementary material for: A Critical View of the Application of the APEX Software (Aqueous Photochemistry of Environmentally-Occurring Xenobiotics) to Predict Photoreaction Kinetics in Surface Freshwaters
Source: Molecules. 2019 Dec 18;25(1):9. doi: 10.3390/molecules25010009 (PMC7017383; doi:10.3390/molecules25010009)
Supplement: Supplementary file 1 [file molecules-25-00009-s001.zip › 3.2.4_gcc-4.4.0.zip!/3.2.4_gcc-4.4.0/3.2.4_gcc-4.4.0/doc/octave/html/interpreter/Executable-Octave-Programs.html]

Executable Octave Programs - Untitled


Next: Comments,
Previous: Errors,
Up: Getting Started


---

### 2.6 Executable Octave Programs

Once you have learned Octave, you may want to write self-contained
Octave scripts, using the #! script mechanism. You can do this
on GNU systems and on many Unix systems ^1^.

Self-contained Octave scripts are useful when you want to write a
program which users can invoke without knowing that the program is
written in the Octave language. Octave scripts are also used for batch
processing of data files. Once an algorithm has been developed and tested
in the interactive portion of Octave, it can be committed to an executable
script and used again and again on new data files.

As a trivial example of an executable Octave script, you might create a
text file named hello, containing the following lines:

```
     #! octave-interpreter-name -qf
     # a sample Octave program
     printf ("Hello, world!\n");
```

(where octave-interpreter-name should be replaced with the full
path and name of your Octave binary). Note that this will only work if
#! appears at the very beginning of the file. After making the
file executable (with the `chmod` command on Unix systems), you can
simply type:

```
     hello
```

at the shell, and the system will arrange to run Octave as if you had
typed:

```
     octave hello
```

The line beginning with #! lists the full path and filename of an
interpreter to be run, and an optional initial command line argument to
pass to that interpreter. The operating system then runs the
interpreter with the given argument and the full argument list of the
executed program. The first argument in the list is the full file name
of the Octave executable. The rest of the argument list will either be
options to Octave, or data files, or both. The -qf options are
usually specified in stand-alone Octave programs to prevent them from
printing the normal startup message, and to keep them from behaving
differently depending on the contents of a particular user's
~/.octaverc file. See Invoking Octave from the Command Line.

Note that some operating systems may place a limit on the number of
characters that are recognized after #!. Also, the arguments
appearing in a #! line are parsed differently by various
shells/systems. The majority of them group all the arguments together in one
string and pass it to the interpreter as a single argument. In this case, the
following script:

```
     #! octave-interpreter-name -q -f # comment
```

is equivalent to typing at the command line:

```
     octave "-q -f # comment"
```

which will produce an error message. Unfortunately, it is
not possible for Octave to determine whether it has been called from the
command line or from a #! script, so some care is needed when using the
#! mechanism.

Note that when Octave is started from an executable script, the built-in
function `argv` returns a cell array containing the command line
arguments passed to the executable Octave script, not the arguments
passed to the Octave interpreter on the #! line of the script.
For example, the following program will reproduce the command line that
was used to execute the script, not -qf.

```
     #! /bin/octave -qf
     printf ("%s", program_name ());
     arg_list = argv ();
     for i = 1:nargin
       printf (" %s", arg_list{i});
     endfor
     printf ("\n");
```

---

#### Footnotes

[1] The #!
mechanism works on Unix systems derived from Berkeley Unix, System V
Release 4, and some System V Release 3 systems.

---
